# Comparative transcriptomic analysis delineates adaptation strategies of *Rana kukunoris* toward cold stress on the Qinghai-Tibet Plateau

**DOI:** 10.1186/s12864-024-10248-8

**Published:** 2024-04-12

**Authors:** Tao Zhang, Lun Jia, Zhiyi Niu, Xinying Li, Shengkang Men, Lu Jiang, Miaojun Ma, Huihui Wang, Xiaolong Tang, Qiang Chen

**Affiliations:** 1 Department of Animal and Biomedical Sciences, School of Life Sciences, Lanzhou University, Lanzhou, China; 2 State Key Laboratory of Herbage Improvement and Grassland Agro-ecosystems, College of Ecology, Lanzhou University, Lanzhou, China; 3 School of Stomatology, Lanzhou University, Lanzhou, China

**Keywords:** Pacbio sequel, Freeze exposure, Hibernation, Emergence period, Liver, Muscle, Tissue specificity

## Abstract

**Background:**

Cold hardiness is fundamental for amphibians to survive during the extremely cold winter on the Qinghai-Tibet plateau. Exploring the gene regulation mechanism of freezing-tolerant *Rana kukunoris* could help us to understand how the frogs survive in winter.

**Results:**

Transcriptome of liver and muscle of *R. kukunoris* collected in hibernation and spring were assisted by single molecule real-time (SMRT) sequencing technology. A total of 10,062 unigenes of *R. kukunoris* were obtained, and 9,924 coding sequences (CDS) were successfully annotated. Our examination of the mRNA response to whole body freezing and recover in the frogs revealed key genes concerning underlying antifreeze proteins and cryoprotectants (glucose and urea). Functional pathway analyses revealed differential regulated pathways of ribosome, energy supply, and protein metabolism which displayed a freeze-induced response and damage recover. Genes related to energy supply in the muscle of winter frogs were up-regulated compared with the muscle of spring frogs. The liver of hibernating frogs maintained modest levels of protein synthesis in the winter. In contrast, the liver underwent intensive high levels of protein synthesis and lipid catabolism to produce substantial quantity of fresh proteins and energy in spring. Differences between hibernation and spring were smaller than that between tissues, yet the physiological traits of hibernation were nevertheless passed down to active state in spring.

**Conclusions:**

Based on our comparative transcriptomic analyses, we revealed the likely adaptive mechanisms of *R. kukunoris*. Ultimately, our study expands genetic resources for the freezing-tolerant frogs.

**Supplementary Information:**

The online version contains supplementary material available at 10.1186/s12864-024-10248-8.

## Background

Understanding how amphibians, especially those living at high altitudes, withstand low temperatures during hibernation can help us better comprehend the adaptation of poikilotherms to extreme conditions.

The behavior and physiological mechanisms of amphibian overwintering have been studied, especially for *Rana sylvatica* (recently renamed *Lithobates sylvaticus*) in North America and *Rana arvalis* in Europe in recent years. Besides the behavioral actions, physiological tolerance is also important for amphibians in cold regions. Ectotherms tolerate subzero body temperatures mainly in two ways, freeze avoidance and freeze tolerance. The painted turtle *Chrysemys picta* [1], the Yarrow’s spiny lizard *Sceloporus jarrovi* [2], the Italian wall lizard *Podarcis sicula* [3], the lizard *Cnemidophorus sexlineatus* [4], and *Lacerta vivipara* [5] have been identified as species [6] that exhibit freeze avoidance thus far. Freeze-avoiding ectotherms must be free of strong ice-nucleating agents (INAs) that can arrange water molecules into a crystalline structure [7]. Some of them produce antifreeze proteins (AFPs) to effectively inhibit inoculation icing [8]. However, overwintering in a supercooled state is a dangerous strategy that can result in death if certain parameters are not satisfied and freezing occurs.

Most freeze-tolerant ectotherms such as insects, intertidal invertebrates, tiny soil invertebrates, amphibians, and reptiles, begin to freeze at high subzero temperatures, apparently to allow gradual and controlled ice development and cell dehydration [9]. When wood frogs (*R. sylvatica*) freeze, it can spend up to 12 h for maximal ice content to be successful, giving ample time for the synthesis and distribution of glucose cryoprotectant and freeze-responsive proteins [10, 11]. Freeze-tolerant species are capable to survive at lower temperature than freeze-avoiding ones. *Upis ceramboides*, an insect from interior Alaska, can withstand temperatures of -60 °C [12]. Winter *R. sylvatica* can survive freezing at -16℃ [13]. *R. arvalis* from Russia tolerate freezing down to -12 or -16 °C, whereas frogs from Denmark survived freezing only to -4 °C [14]. Freeze-tolerant species may withstand 50 ∼ 65% of their body fluid as extracellular ice for weeks or months while still maintaining normal states after thawing [15]. However, some species, e.g. *C. picta* and *L. vivipara*, could alternate between the reciprocally sole strategies of freeze avoidance and freeze tolerance [16].

Both freeze-avoiding and freeze-tolerant ectotherms accumulate cryoprotectants before winter comes. In freeze-tolerant species, by colligatively depressing the FP of body fluids, these solutes permit the cytoplasm to remain super cooled whilst the extra cellular fluids freeze, and also limit ice formation [17]. Furthermore, many cryoprotectants function in antioxidation, energy supply, macromolecular stabilization, and counteraction of perturbing solutes [18]. Cryoprotectants found in amphibians include carbohydrates and even the metabolic waste urea [18]. Urea serves as a cryoprotectant in *Nanorana pleskei* which is indigenous to the northeastern Qinghai-Tibetan Plateau [19].

Despite the fact that melting and icing can be fatal, a variety of molecular and physiological reactions can minimize the harm they do to cells and tissues. Nine glycolytic enzymes and three urea cycle enzymes were differentially phosphorylated in liver of *R. sylvatica* under 24 h freezing, 24 h anoxia, and 40% dehydration exposures [20]. *R. sylvatica* showed significant changes in the phospholipid composition and lipid ratios of hepatocyte membranes. Less cholesterol [21] and most notably an increase in phosphatidylethanolamine may help to maintain fluidity at low temperatures [22].

Numerous novel potential targets sensitive to freezing were discovered by genomic and proteomic studies [15]. Through analysis of the hepatic transcriptome of *Dryophytes chrysoscelis*, various genes associated with the ubiquitin proteasome system, DNA repair, and the heat shock protein response were shown to be increased in cold exposed and frozen individuals [23].

Both the European *Rana lessonae* [24] and the Alaskan *R. sylvatica* [25] have antifreeze glycolipids (AFGL), which have comparable action to AFPs. AFGLs are positioned on cell membranes to prevent inoculation of the cytosol and recrystallization in freeze-tolerant species [8]. Freeze-responsive protein 10 (Fr10) found in *R. sylvatica* showed extremely dynamic expression in response to seasonal freezing stress and its overexpression can improve cellular freezing resistance [26]. The novel protein was identified to have ice recrystallization inhibition (IRI) activity. By using the SPLAT cooling assay, it was directly seen that the average grain size of ice crystals decreased by 40% in the presence of 30 µM of Fr10 compared to the control samples [27]. Another novel freeze-responsive gene (Li16) was discovered in the liver of *R. sylvatica* [28, 29]. The 12-kDa type IV fish AFP (LS-12) was discovered in longhorn sculpins (*Myoxocephalus octodecimspinosis*), which differs structurally from other fish AFP types and share little amino acid sequence with Fr10 [30].

*Rana kukunoris*, a close relative to *R. sylvatica*, is distributed in the east Qinghai-Tibet Plateau and nearby areas at the altitude of 2000–4400 m, and the frog plays an important role in the local food chain and ecosystem. In the northeast of Qinghai-Tibet Plateau, winter temperatures can drop below − 20 °C and the soil temperature can be as low as -7 °C at 20 cm depth.

In contrary to the intensive study on its relative *R. sylvatica*, the overwintering mechanism of *R. kukunoris* has not been studied. What tactic did the frogs employ to survive the winter? In this work the fluctuant gene expression during and after the hibernation will be examined to reveal possible critical genes for effective overwintering of *R. kukunoris* at the transcriptome level. We try to figure out what cryoprotectants are used, whether ice-binding proteins are produced, and the roles these substances played in the adaptation to cold environment. This will aid in the advancement of animal ecophysiology and offer the necessary building blocks for the preservation of amphibians, the Plateau ecology, and even the viability of transplantable human cells and organs after thawing.

## Results

When dug out, hibernating frogs were corporeal frozen with rigid limbs, tightly closed eyelids, visible signs of dehydration, and ice particles around the body. Three of six partly frozen frogs recovered when exposed to room temperature.

### Quality of transcriptome sequencing data, annotation and differentially expressed genes

The RNA integrity number (RIN) value was 7.8 in mixed twelve samples sequenced in Pacific Bioscience (PacBio) Sequel platform. Using Single Molecule Real-Time (SMRT) sequencing technology from PacBio, a full-length transcriptome of *R. kukunoris* was produced. The sequencing results generated 51.23 GB (25,497,349 reads) subreads data with average subreads length of 2010 bp and N50 length of 2195 bp (Table S1). A total of 10,062 unigenes were obtained, and 57,446 coding sequences (CDS) was found within transcripts.

By combining protein data from five different species, we were able to create a reference dataset representing the main lineages of amphibian species which updated the genome assembly using the latest sequencing methods. After cut-off for identity (greater than 80% threshold) and E-value, 9,924 CDSs of *R. kukunoris* were successfully annotated. 9,373 annotated CDSs had an E-value below 1E-50.

A total of 93,431,337,600 raw base pair (bp) which range from 6,390,937,800 to 9,972,295,800 were generated for the twelve samples. The RIN values were all greater than 7.6. After filtering, 586,884,222 reads which range from 39,887,398 to 62,964,078 were retained. Percentage of each sample with base greater than Q30 (phred quality score; Q score) were all above 91.31. Read mapping ratios of twelve sequence data ranged from 64.06 to 80.05% (Table S2).

**Fig. 1:**
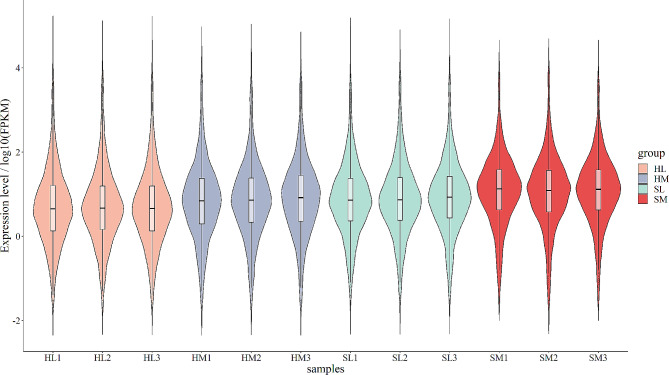
Expression level of each sample. Expression level, shown as the fragments per kilobase million (FPKM) in y-axis, is plotted on a log10 scale. The number of genes at a certain expression level is represented by the breadth of the violin plot

**Fig. 2:**
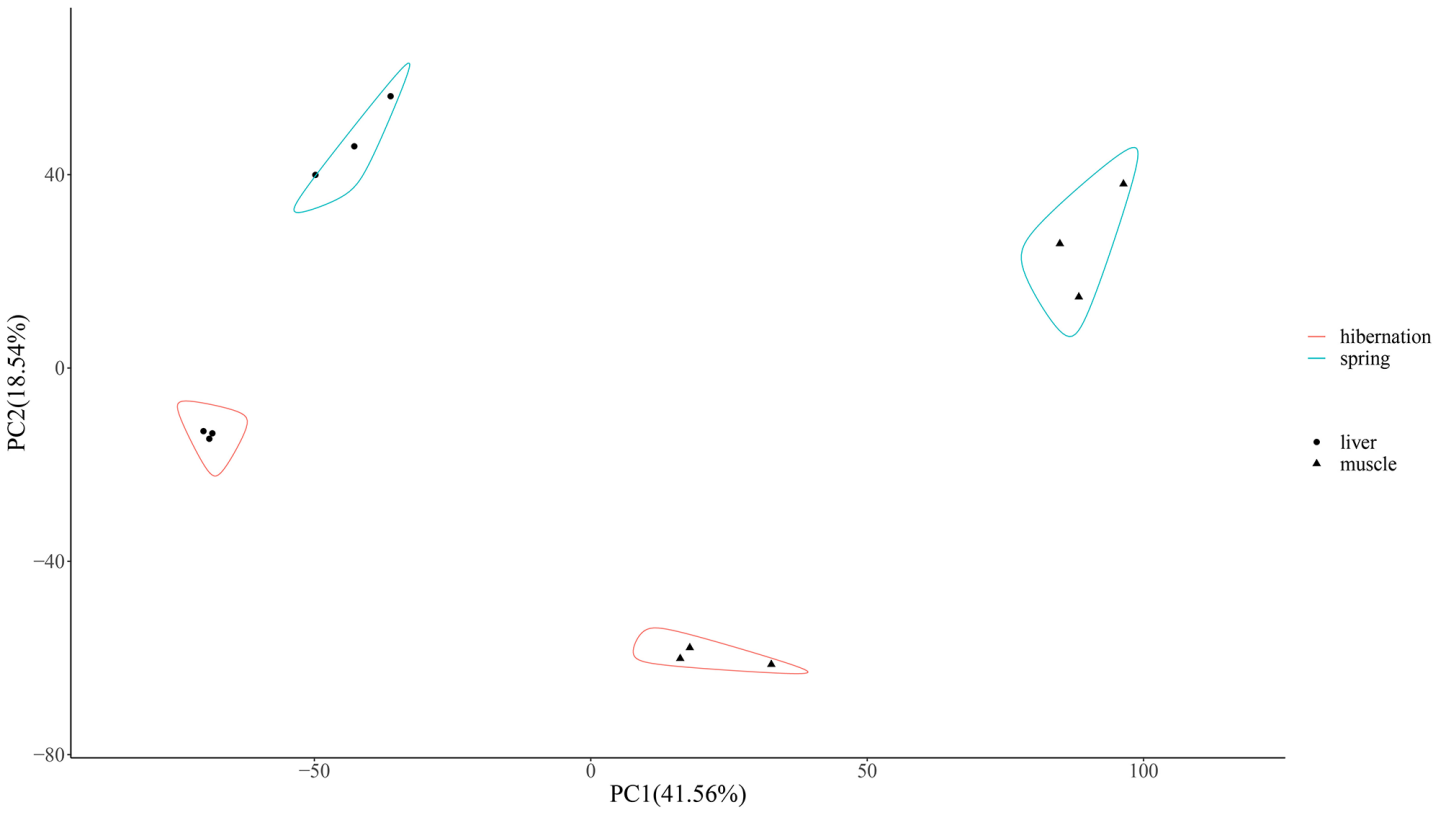
Principal components analysis (PCA) of gene expression of liver (circles), muscle (triangles), hibernation (red lines) and spring (cyan lines). The expression values, in terms of fragments per kilobase million (FPKM), were used as the input for PCA

**Fig. 3:**
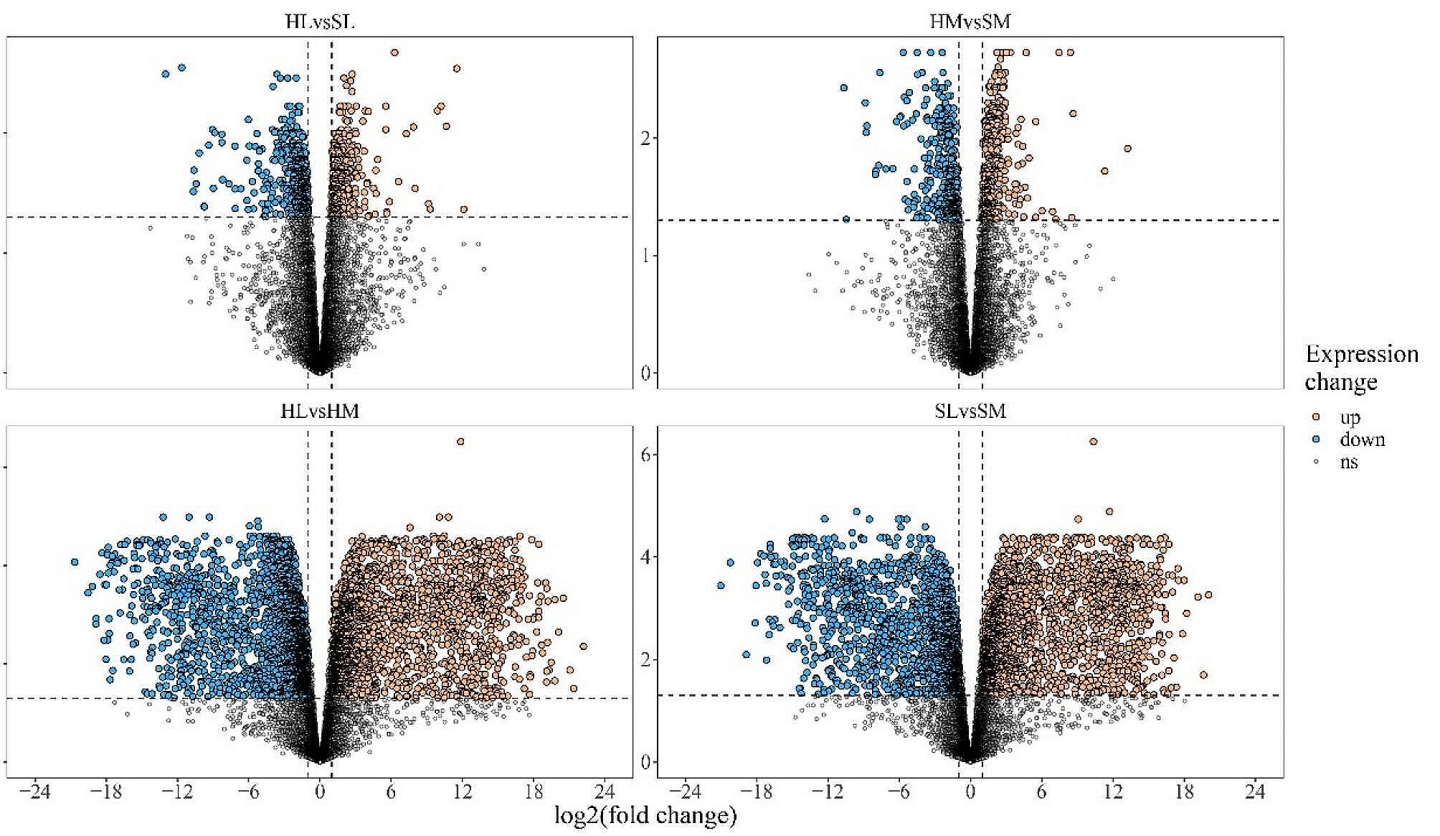
Volcano plot depicting transcriptomic change in seasons and tissues. Horizontal dotted line represents the adjust P-value = 0.05, and vertical dotted lines represent the absolute value of log2(fold change) = 1. The number of differentially expressed genes in each group compared with spring or muscle. liver (L), muscle (M), hibernation (H) and spring (S). HL vs. SL, HM vs. SM, HL vs. HM, SL vs. SM

**Fig. 4:**
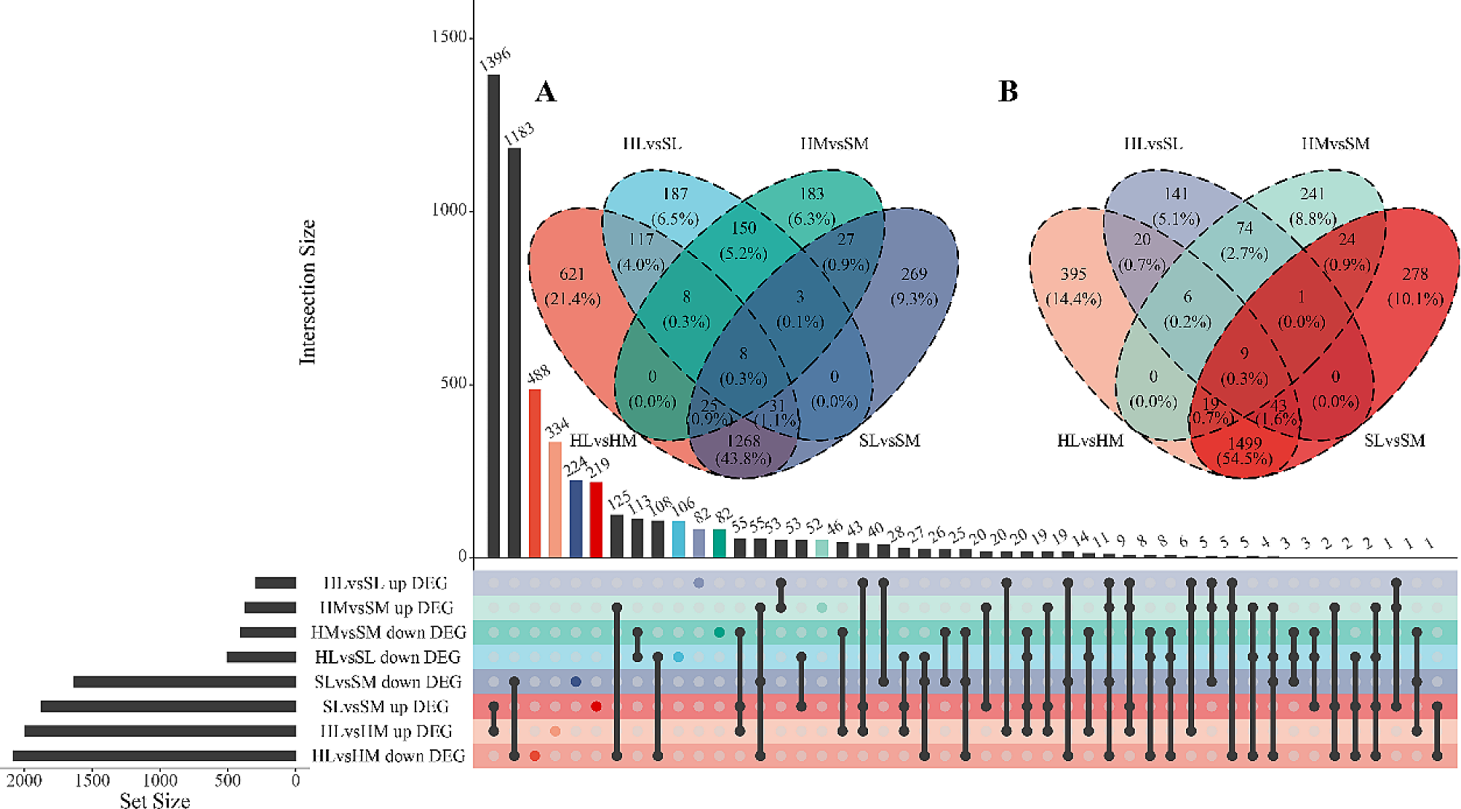
Visualizations of DEG (differential expression gene) intersecting sets through Venn and UpSet plot. **A**: Venn plot of down-regulated DEGs. **B**: Venn plot of up-regulated DEGs. The color of each group matches the one that is displayed in the lower left corner

Each sample has the roughly equal level of overall RNA expression (Fig. 1). We evaluated the differences of gene expression between seasons (hibernation and spring) and tissues (liver and muscle) through principal components analysis (PCA). The first component explained 41.56% of the variance and the second explained 18.54% (Fig. 2). With samples of muscle and spring as reference group, 798 DEGs (504 down, 294 up) in HL vs. SL group, 778 DEGs (404 down, 374 up) in HM vs. SM group, 3504 DEGs (1631 down, 1873 up) in SL vs. SM group, and 4069 DEGs (2078 down, 1991 up) in HL vs. HM group were identified respectively (Figs. 3 and 4, Table S3). Detail information of differentially expressed genes (DEGs) in four groups was showed in Table S4.

### The transcript expression of antifreeze proteins

**Fig. 5:**
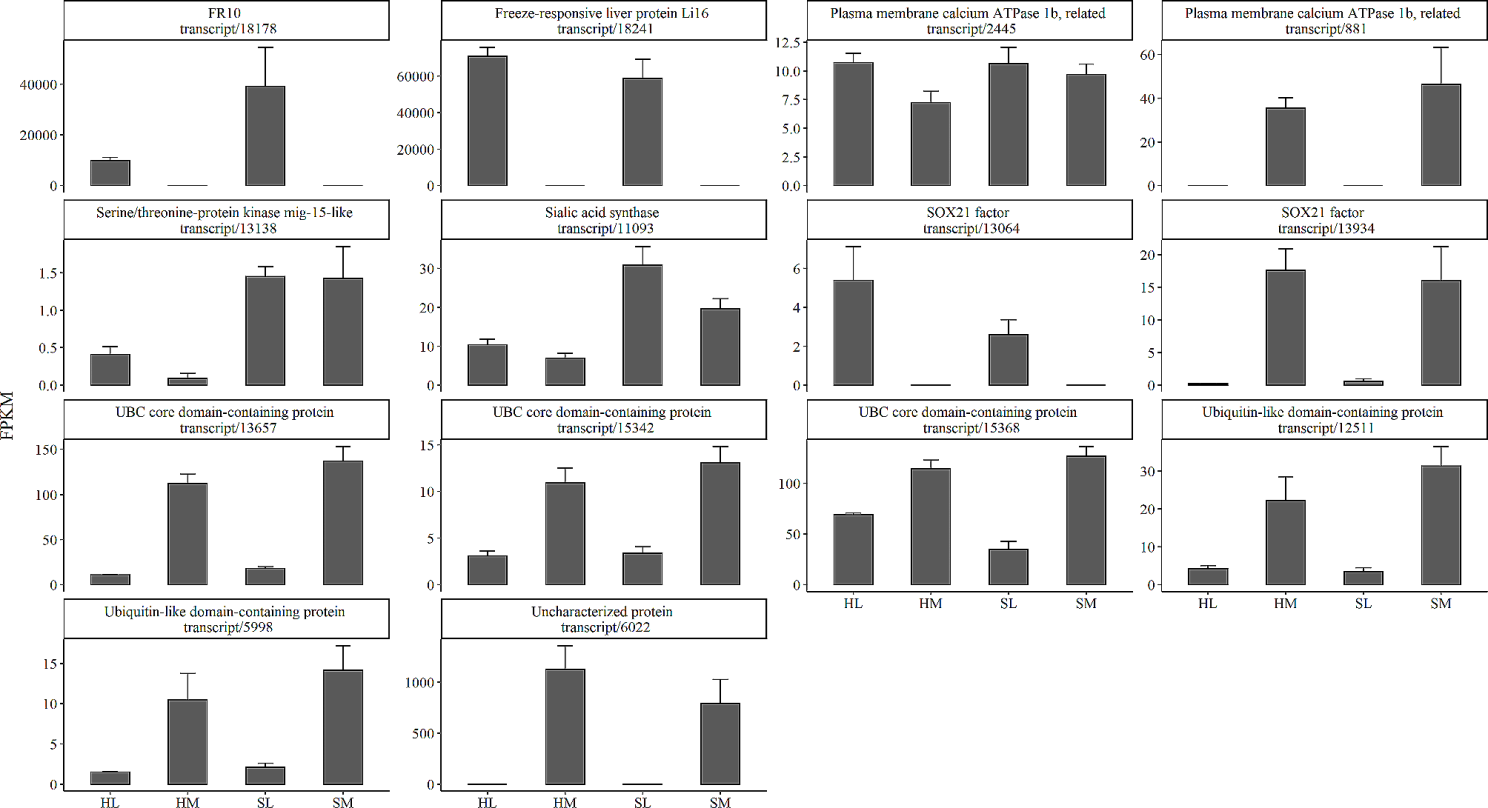
Expression histogram of AFP expression genes in tissue and season of *Rana kukunoris*. Text on the top side of subgraph is composed of the protein name and the transcript ID in sequence file. liver (L), muscle (M), hibernation (H) and spring (S)

The outcomes of antifreeze protein dataset searches were filtered for identity (greater than 60% threshold) and E-value, and 18 significant DEGs of 48 isoform AFPs were identified to nine putative AFPs in the *R. kukunoris* (Fig. 5, Table S5). Three-dimensional homology modeling was performed by AlphaFold to infer the tertiary structure of the putative AFPs (Supplementary Data 1 ). Most protein models have alpha helices and beta sheet. The predicted LDDT (pLDDT) scores of these models are all above 74%, range from 74.3 to 96.4%, except three models (FR10 and two isoforms of SOX21 factor, range from 55.5 to 61.5%). The source species of the nine putative AFPs are *Acipenser ruthenus* (sterlet sturgeon), *Nicator chloris*, *Hirondellea gigas*, *Tetradesmus obliquus* (*Acutodesmus obliquus*, green alga), *Eimeria praecox*, *Aureobasidium pullulans* EXF-150, *Spirometra erinacei* (*Spirometra erinaceieuropaei*, tapeworm), and *R. sylvatica* (*L. sylvaticus*, wood frog). The putative AFPs *FR10* (83.3% identities), *Li16* (83.3% identities) and uncharacterized/hypothetical protein (uniprot ID: A0A7M3QFV7, 100% identities) are noteworthy.

### Notable genes with interest

Five glucose transporter (GLUT) genes were significantly expressed in the four groups. Muscles have higher levels of GLUT4 expression than livers. The increased expressions of GLUT5, GLUT6, GLUT9 and glucose-6-phosphate transporter (G6PT1) were observed in liver. A rise in GLUT8 expression was found in muscle and liver of spring frogs. In addition, the expression of glucose-6-phosphatase (G6PC), UDP-glucose 4-epimerase (GALE) and UDP-glucose 6-dehydrogenase (UGDH) were elevated in SL group. The expression of glucose-6-phosphate isomerase (GPI) and UTP-glucose-1-phosphate uridylyltransferase (UGP2) were super-elevated in HM group (Supplementary Fig. 1).

The expression of ornithine carbamoyltransferase (OTC, argF, argI), carbamoyl-phosphate synthase (CPS1) and arginase (rocF, arg, ARG) were relatively high in HL and SL groups in urea cycle (Supplementary Fig. 2). The expression of sn1-specific diacylglycerol lipase (DAGL), hepatic triacylglycerol lipase (LIPC) and 2-acylglycerol O-acyltransferase 2 (MOGAT2) were high in liver. The expressions of certain genes were soared in liver or muscle, such as glycerol-3-phosphate O-acyltransferase (GPAT), glycerol kinase (GK), diacylglycerol kinase (DGK), sn1-specific diacylglycerol lipase (DAGL), diacylglycerol cholinephosphotransferase (CPT1) and diacylglycerol O-acyltransferase 1 (DGAT1) (Supplementary Fig. 3). Additionally, the expression of glycerol-3-phosphate dehydrogenase (glpD) was low in liver.

### KEGG terms enriched of differentially expressed gene

A positive z-score value indicates the genes is upregulated, while a negative z-score value indicates the genes is downregulated in a pathway or term.

#### Inter-seasonal variations

**Fig. 6:**
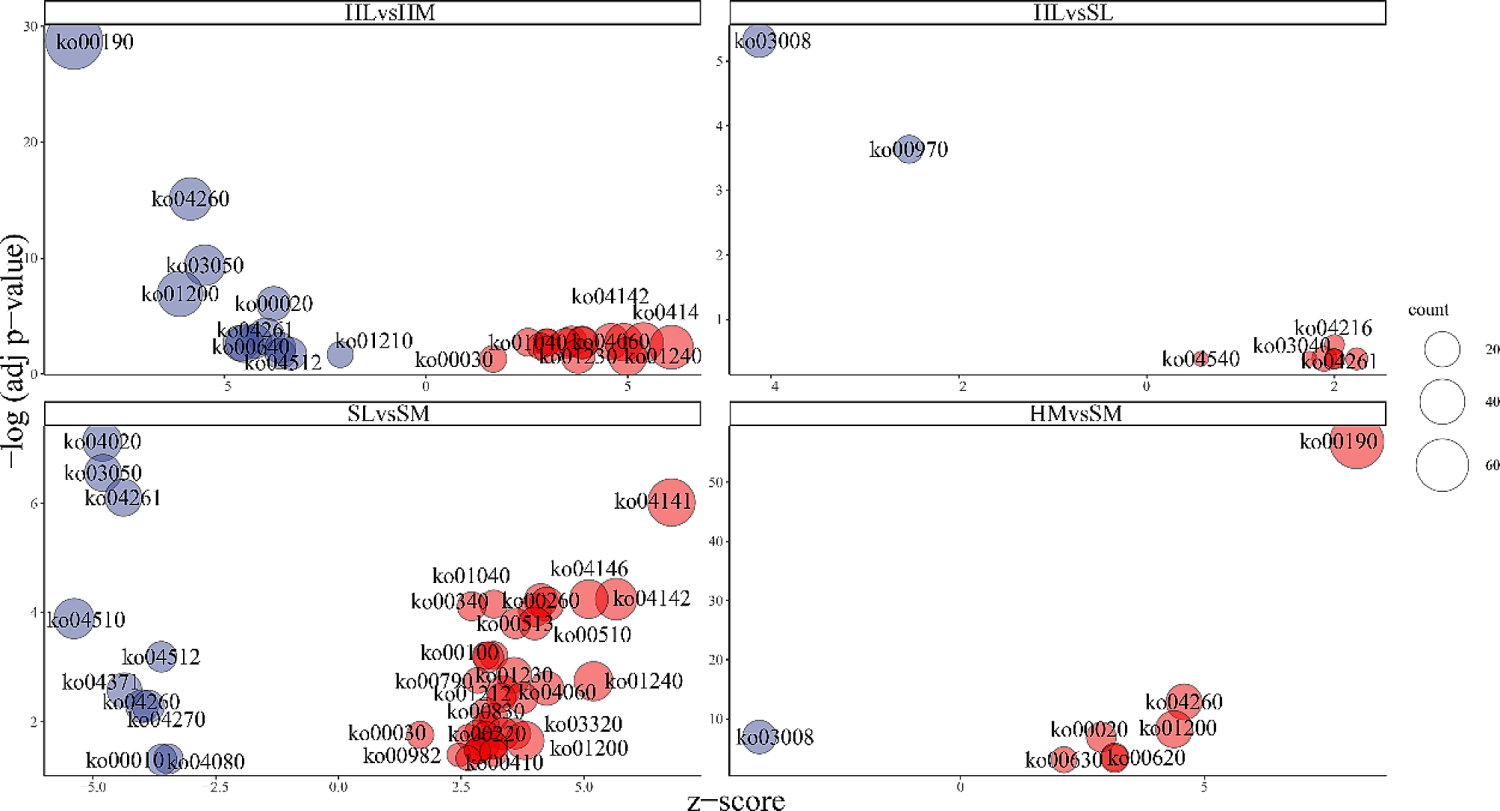
Overview bubble plots of KEGG (Kyoto Encyclopedia of Genes and Genomes) enriched terms. The z-score is assigned to the x-axis and the negative logarithm of the adjusted p-value to the y-axis. The area of the displayed circles is proportional to the number of genes assigned to the term and the color corresponds to the down-regulated terms (blue) or up-regulated terms (red). liver (L), muscle (M), hibernation (H) and spring (S)

KEGG enrichment analysis showed that totally 57 KEGG pathways were significantly enriched (BH-adjusted p-values < 0.05) in four groups (Fig. 6, Table S6). The ribosome biogenesis in eukaryotes and aminoacyl-tRNA biosynthesis were notably suppressed in HL vs. SL and HM vs. SM. The up-regulated KEGG pathways in HM vs. SM include “Oxidative phosphorylation”, “Cardiac muscle contraction”, “Carbon metabolism”, “Citrate cycle (TCA cycle)”, “Pyruvate metabolism”, “Glycolysis/Gluconeogenesis”, and “Glyoxylate and dicarboxylate metabolism”.

#### Differences between tissues

Comparison of HL vs. HM and SL vs. SM jointly yielded six pathways with negative z-score values, including “Calcium signaling pathway”, “Proteasome”, “Adrenergic signaling in cardiomyocytes”, “ECM-receptor interaction”, “Cardiac muscle contraction” and “Glycolysis/Gluconeogenesis”. Also with negative z-score values, seven pathways chiefly related to carbohydrate, energy and amino acid metabolism were displayed in HL vs. HM; four pathways related to signal transduction, signaling molecules and interaction, cellular community and circulatory system were discovered in SL vs. SM.

Sixteen pathways with positive z-score values yielded by HL vs. HM and SL vs. SM are “Cytokine-cytokine receptor interaction”, “Various types of N-glycan biosynthesis”, “Peroxisome”, “N-Glycan biosynthesis”, “Biosynthesis of unsaturated fatty acids”, “Histidine metabolism”, “One carbon pool by folate”, “Lysosome”, “Glycine, serine and threonine metabolism”, “Sphingolipid metabolism”, “Folate biosynthesis”, “Protein processing in endoplasmic reticulum”, “Steroid biosynthesis”, “Biosynthesis of amino acids”, “Biosynthesis of cofactors” and “Pentose phosphate pathway”. These mainly related to “Carbohydrate metabolism”, “Lipid metabolism”, “Amino acid metabolism”, “Glycan biosynthesis and metabolism”, “Metabolism of cofactors and vitamins”, “Folding, sorting and degradation”, “Signaling molecules and interaction” and “Transport and catabolism”. The 23 significant findings of SL vs. SM are predominantly related to “Carbohydrate metabolism”, “Lipid metabolism”, “Amino acid metabolism”, “Metabolism of other amino acids”, “Metabolism of cofactors and vitamins”, “Xenobiotics biodegradation and metabolism”, “Folding, sorting and degradation”, “Cell growth and death” and “Endocrine system”; No specific outcomes of HL vs. HM emerged from the KEGG pathway enriched analysis.

### Gene ontology terms enriched of differentially expressed gene

#### Inter-seasonal variations

**Fig. 7:**
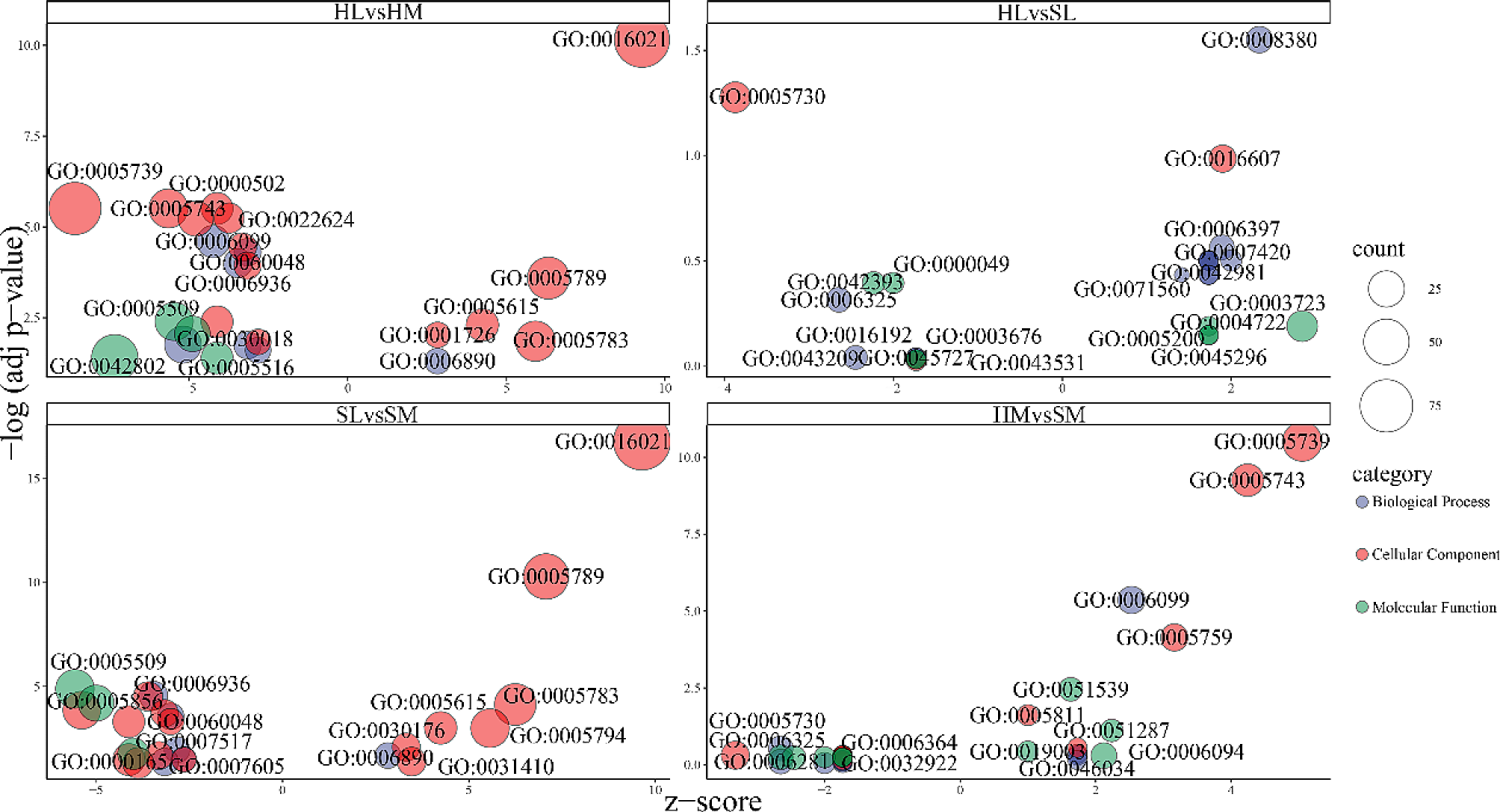
Overview bubble plots of GO (Gene Ontology) enriched terms. The z-score is assigned to the x-axis and the negative logarithm of the adjusted p-value to the y-axis. The area of the displayed circles is proportional to the number of genes assigned to the term and the color corresponds to the category (biological process, cellular component, molecular function). liver (L), muscle (M), hibernation (H) and spring (S)

Gene set enrichment analysis was performed based on the GO molecular function, biological process, and cellular component classifications. In total, 37 GO terms are significantly enriched (BH-adjusted p-value < 0.05) (Fig. 7, Table S7). One significantly up-regulated term was RNA splicing in HL vs. SL. Six significantly up-regulated terms in HM vs. SM are “tricarboxylic acid cycle”, “lipid droplet” and “4 iron, 4 sulfur cluster binding”, other three terms centered around mitochondrion, including “mitochondrion”, “mitochondrial inner membrane”, and “mitochondrial matrix”. No term was remained in down-regulated DEGs of HL vs SL or HM vs SM after filtering by BH-adjusted p-value < 0.05.

#### Differences between tissues

The five pathways of up-regulated DEGs in HL vs. HM and SL vs. SM are “retrograde vesicle-mediated transport, Golgi to endoplasmic reticulum”, “integral component of membrane”, “endoplasmic reticulum membrane”, “extracellular space” and “endoplasmic reticulum”; the three significantly up-regulated GO terms of SL vs. SM specific results are “Golgi apparatus”, “integral component of endoplasmic reticulum membrane” and “cytoplasmic vesicle”. It should be noted that membrane and protein synthesis functions were enriched. Only one cellular component term (ruffle) emerged from HL vs. HM specific outcomes.

The eleven pathways of down-regulated DEGs in HL vs. HM and SL vs. SM are “muscle contraction”, “cardiac muscle contraction”, “muscle organ development”, “proteasome accessory complex”, “myosin II complex”, “Z disc”, “myofibril”, “proteasome complex”, “calcium ion binding”, “actin filament binding” and “calmodulin binding”. These mutual GO terms showed a high skew toward terms regarding to muscle function. The six significantly down-regulated GO terms of SL vs. SM specific results are “sensory perception of sound”, “MAPK cascade”, “cytoskeleton”, “extracellular region”, “cilium” and “actin cytoskeleton”. Eight GO terms which contain “tricarboxylic acid cycle”, “ubiquitin-dependent protein catabolic process”, “glycolytic process”, “mitochondrion”, “mitochondrial inner membrane”, “mitochondrial matrix”, “proteasome core complex” and “identical protein binding”, emerged from HL vs. HM specific outcomes.

## Discussion

In present study we used next-generation sequencing (NGS) and single molecule real-time (SMRT) sequencing to look at gene expression in liver and muscle tissue of the hibernation and spring frogs. Our examination of the mRNA responding to whole body freezing and recover revealed the key genes and pathways associated with freeze-induced responses in *R. kukunoris*. Functional pathway analyses point toward differential regulation of transcript expression with regard to antifreeze protein, cryoprotectant, ribosome, energy supply and protein metabolism.

### General overview of transcript expression results

The full-length transcriptome data can offer more information for further exploration and improving genomic data of *R. kukunoris*. Principal components analysis (PCA) demonstrated distinct intergroup differences and excellent intragroup biological replication. More DEGs and more enriched pathways were found in comparison between tissues than between seasons. This indicates that the liver and muscles may take substantially varied response to the seasonal change of environment and certain physiological processes active in winter may reserve in spring.

### The mRNA expression of AFPs varies according to the species and tissue

High predicted LDDT (pLDDT) scores of three-dimensional structural models of AFPs indicate high confidence residues in the proteins. The identity of deduced AFPs to reference AFP was greater than 60% threshold suggesting the AFP is conservative. The same transcript contains multiple identical AFP-encoding sequences and different transcripts encode the same AFP. This can maintain the organism’s AFP level and ensures its ability to withstand freezing.

Two of the three transcripts of *UBC* (ubiquitin-conjugating domain core domain-containing) gene contain significant variations of expression in both tissue comparisons. There were continuous expressions of all AFPs in both winter and spring. This indicated the spring frogs retain their wintertime resistance to freezing in order to deal with the unpredictable springtime temperatures on the Qinghai-Tibet plateau. Monthly minimum air temperature was − 2.5±0.7 °C in April in Maqu County from 2005 to 2019 according to China meteorological data service center (National meteorological information center).

Exceptionally high expressions of *FR10* [27, 31, 32] and *Li16* [28] genes were found in the liver of hibernation and spring *R. kukunoris*, however, there was scarce expression in muscle. Nevertheless, another well-known freeze tolerance-associated gene *FR47* which was highly expressed in the livers of *R. sylvatica* [33] was not discovered in *R. kukunoris* in this study. Genes of ubiquitin-like domain-containing protein and uncharacterized/hypothetical protein were high expressed only in muscle of *R. kukunoris*. These denote that the expression of AFP genes was tissue-specific and depend on species.

### Production-related genes for urea and glucose were actively deployed

Our previous studies have shown that large quantities of glycogen and glucose were determined in the muscles of the summer and fall *R. kukunoris* from Maqu after cold acclimation. In addition, for *R. kukunoris* obtained in the fall, the amount of glucose in the liver increased 31-fold after freezing, and urea content in the liver also dramatically increased after cold acclimation [34]. G6PT1 [35] and G6PC [36] were evidently highly expressed in the liver but not in the muscle of *R. kukunoris*. In vertebrates, glucose 6-phosphate (G6P) was transported by G6PT1 to the endoplasmic reticulum, where G6PC then dephosphorylated it. The significantly higher expression of G6PT1 and G6PC may result in large quantity of glucose buildup as cryoprotectant. Although glucose protect the body against freezing, excessive levels of glucose may have the deleterious consequence of glycating biomolecules [15]. High level expressions of specific GLUTs were determined in all of our four groups. Active GLUTs may regulate the concentration of intracellular and extracellular monosaccharides and prevents the negative consequences of glycation. Although glucose can be produced by the liver from amino acids, it may be taken into muscles with the aids of GLUTs, where it was quickly converted to muscle glycogen, avoiding the risk of glycation of biological macromolecules. GLUT4 was also found to be expressed in skeletal muscle of cold-hardy frog *Rana dybowskii* in winter [37]. GLUT8 mRNA levels in the liver of the freeze-tolerant Cope’s gray treefrog *D. chrysoscelis* reduced following freezing [23]. This implicates the importance of GLUTs in the cold-hardy ectotherms.

Three genes of central urea cycle, *CPS1*, *OTC*, and *ARG* (arginase, responsible for the last step to produce urea) were highly expressed in the liver. This may lead to high level of urea accumulating acting as cryoprotectant in *R. kukunoris*. It is reported that urea also contributes to the anti-freeze protection for *R. sylvatica* [38], *D. chrysoscelis* [23, 39] and *N. pleskei* [19].

It is documented that glycerol is the primary anti-freeze protectant for *Dryophytes versicolor* (before named *Hyla versicolor*) [40] and *D. chrysoscelis* [39, 41], despite not being the most cost-effective. However, in *R. kukunoris* glycerol may not act as cryoprotectant beside glucose and urea. Our results showed that the glycerol-depleting genes (*GPAT*, *GK*, *DGK*, *DAGL*, *CPT1* and *DGAT1*) were strongly expressed in the liver or muscle indicated that the frog may consume glycerol to synthesize phospholipids which were needed to maintain the integrity of the membrane, and as a substrate to produce triacylglycerol.

### The hibernating frogs continued to synthesize proteins

The mRNA involved in ribosome and aminoacyl-tRNA biogenesis was detected in the liver of hibernating *R. kukunoris* indicating that the protein synthesis with high energy expenditure still maintained. Based on the metabolic rate depression theory (MRD) [9, 15], the hibernating frogs should not require a lot of proteins to sustain normal metabolic activity. However, to guarantee the production of crucial proteins like AFP, many genes involved in protein synthesis may be also required to be expressed at a certain level (Supplementary Fig. 4). Our results showed elevated expression of genes related to ribosome and aminoacyl-tRNA biogenesis in spring frogs compared to hibernation. Large quantity of fresh, healthy, effectively functioning proteins is needed in the spring to support metabolism for activities including locomotion, foraging, digesting, reproduction, and cardiopulmonary resuscitation. Liver microRNA transcriptomics revealed that accelerated ribosome synthesis and inhibition of energy-expensive pathways occurred in *D. versicolor* collected during the spring mating season and artificially frozen in a laboratory [42]. Aminoacyl-tRNA production was also perhaps one of the most noticeably changed routes in overwintering frogs *N. parkeri*, according to LC-MS analyses [43, 44].

### Energy supply in the muscles of hibernating frogs was not zero

Most of the up-regulated genes were significantly enriched onto energy generation terms in HM vs. SM (Supplementary Fig. 5, Supplementary Fig. 6, Supplementary Fig. 7). In *R. sylvatica*, reduced oxygen causes the genes involved in oxidation in mitochondria to be upregulated in response to freezing, which presumably serves an adaptive purpose in sustaining cellular energetics throughout indefinite periods of whole-body freezing [45]. Anaerobic energy generation in the liver and leg muscles of *D. versicolor* were possibly necessary for survival under freezing [46]. It is important to remember that the muscle need to maintain a low level (not zero) of metabolism so that it can carry out essential processes like glucose transport. It has been shown that the intracellular water of freezing-tolerant ectotherms does not freeze when the body freezes [47] indicating that the cells are still engaged in some rate of metabolic activity. Long-term hypoxia improved the affinity for oxygen of isolated mitochondria from the skeletal muscle of hibernating *R. temporaria* [48]. In addition, our results showed that amino acid was catabolized for energy in the fasting HM group (Supplementary Fig. 8). It is not surprising since fasting animals usually metabolize protein to produce energy. The tadpoles at metamorphic climax may also experience the same limit of energy as hibernation. Amino acid contributed more to energy supply in *R. omeimontis* tadpoles than in pre-metamorphic ones [49]. Similarly, compared to control turtles, the total blood amino acid pool of the freezing-exposed hatchlings of the painted turtle (*C. picta marginata*) rose 2.25 times [50].

Distinct up-regulation pathways involved in important physiological processes including cell motility, cell proliferation, cell differentiation, cardiovascular functions, and interaction with signaling molecules (Supplementary Fig. 9) were determined in SM vs. SL of our study. This may hasten the recovery of the muscles.

The up-regulation of proteasome, a protein-degrading mechanism, in muscle can eliminate the proteins damaged during hibernation in order to quickly restore activities in spring. This is similar to the finding in *R. sylvatica*, its proteasome selectively destroys oxidatively damaged proteins in muscle during freezing [51].

### Lipid metabolism was enhanced for reproduction in spring

Lipids (glycerolipid, fatty acid, glycerophospholipid and ether lipid) metabolism were accelerated in SL vs. SM but not in HL vs. HM. Spring frogs may predominantly utilize fat for energy. The main reason why spring frogs consume a lot of fat may be that they need more energy for reproduction. The fat body weight of *Rana pipiens* declined to 25.1 mg/100 g in the spring, which was lower than that recorded in the fall and winter [52]. The larger pre-hibernation energy stores satisfy the springtime energy need for reproduction [53]. Fat bodies of *R. kukunoris* were observed to be tiny compared to autumn frogs in our previous anatomical observations.

## Conclusions

Our results showed that *R. kukunoris* possess the ability of freeze tolerance. FR10 and Li16 were highly expressed as the major antifreeze proteins. The analyses of gene expression implicated accumulation of glucose and urea as cryoprotectants. Genes related to energy supply in muscle of winter frogs were up-regulated. Hepatic thriving metabolism fixed the harm caused by hibernation progressively and supported the physiological activities in spring.

## Methods

### Tissue collection

**Fig. 8:**
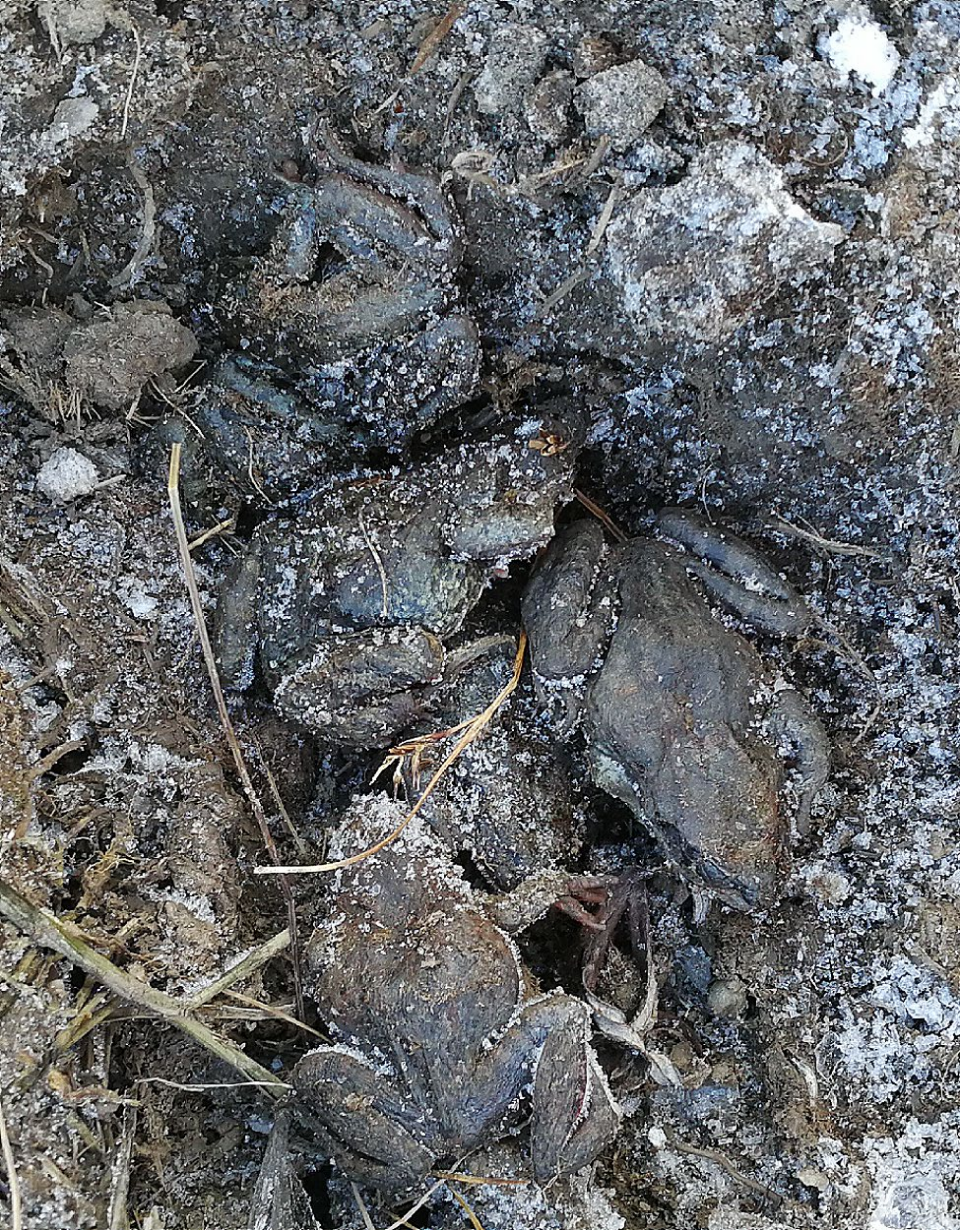
Frogs (*Rana kukunoris*) huddled in the wire fence in hibernation (Date of shooting: November 28, 2021)

We built small puddle (1 × 1 m^2^) with shallow water and grass surrounded by wire fence (1 m high) to simulate the natural environment at Gannan Grassland Ecosystem National Observation and Research Station (33°40′ N, 101°52′ E, altitude 3540 m) in the fall of 2021 in Maqu County, Gansu Province, China. Ten adult frogs (*R. kukunoris*) were placed in the enclosure in mid-September, and six plateau frogs were found huddled under 30 cm soil in the enclosure with ice on their bodies in November 28, 2021 (Fig. 8). The microenvironment temperature was 1.6 °C when the frogs were exposed by excavation. Three frogs (three males) were successfully recovered and three frogs (three females) died. Spring samples were collected from the field near the fence in April 26, 2022. Six male frogs from two groups (three frogs each) of hibernation (H) and spring (S) were quickly sacrificed to collect liver (L) and muscle (M) which then immediately frozen with liquid nitrogen.

### Total RNA extraction, library construction, and transcriptome sequencing and assembly

Liver and muscle samples in two groups of hibernation and spring were transported to Novogene (Beijing, China) with carbon dioxide ice. The integrity, quality and concentration of RNA samples were checked by Agarose gel electrophoresis and Nanodrop 2000. Based on the PacBio Sequel platform [54], all samples were mixed in equal amounts to obtain complete transcripts containing polyA tails directly, and one full-length transcriptome was measured. Twelve second-generation transcriptomes were obtained by Illumina NovaSeq 6000 platform and 150 paired-end reads were generated. Clean data were obtained by removing reads containing adapter and ploy-N and low quality reads from raw data. All the subsequent analyses were conducted using clean, high-quality data.

The subreads were acquired from polymerase reads using the SMRT Link (version6.0; parameter -min_length 50) pipeline supported by PacBio’s official, and Circular Consensus Sequence (CCS) reads were extracted out of subreads’ BAM file. Through IsoSeq3, CCS reads were classified into full-length (FL), full-length non-chimeric (FLNC), non-full-length (NFL) based on cDNA primers and polyA tail signal. Subsequently, the FLNC reads were clustered to generate the cluster consensus isoforms. Finally, the NFL sequence was used to modify the obtained consistent sequence (polished). Above steps were completed according to the official workflow documentation (https://github.com/PacificBiosciences/IsoSeq). Additional nucleotide errors in consensus reads were corrected using the Illumina RNA-seq data with the software LoRDEC [55]. To yield a final set of non-redundant transcript sequences, CD-HIT (version4.8.1; parameter -c 0.95, -G 0, -aL 0.00, -aS 0.99, -AS 30) software was used to merge highly similar sequences and remove redundant sequences from high-quality transcript. The outputted transcript sequences were used as reference set for functional annotation and differential expression analysis, and to predict CDS. TransDecoder (version 5.5.0, -m 10) was used to identify the candidate Coding Sequence (CDS) regions within transcript sequences (https://github.com/TransDecoder/TransDecoder). To further maximize sensitivity for capturing ORFs that may have functional significance, results of blastp search against Uniref90 and hmmscan searching Pfam-A were integrated into coding region selection.

### Functional annotation of full-length transcriptome sequences

Due to the lack of genomic resources of *R. kukunoris*, we combined protein data from five species to create a reference dataset for gene annotation, which includes *Nanorana parkeri*, *Rana temporaria*, *Leptobrachium leishanense*, *X. tropicalis* and *X. laevis*. The complete *X. laevis* and *X. tropicalis* proteomes were downloaded from Uniprot database [56]. The protein data and annotation files of *N. parkeri* and *L. leishanense* were downloaded from figshare (https://figshare.com/projects/Genomic_data_of_Nanorana_parkeri/116061; https://figshare.com/articles/dataset/Genome_assembly_of_Leptobrachium_leishanense/8019986 ), and protein data *R. temporaria* was downloaded from NCBI (National Center for Biotechnology Information). In addition, antifreeze protein dataset was created for special annotation (https://www.uniprot.org/keywords/KW-0047).

AlphaFold2 (version 2.0.0) [57] was used to generate the protein models of all putative AFPs from *R. kukunoris*. A python script (plddt2csv.py) was applied to extract the predicted local distance difference test (pLDDT) value from results of Alphafold2 (https://github.com/CYP152N1/plddt2csv). Open-source PyMOL (version 2.5.0, https://github.com/schrodinger/pymol-open-source) was operated to render models [58]. An open source plugin (alphafold_coloring.py) was implemented in PyMOL to tint AlphaFold protein structure database predictive models by pLDDT confidence score (https://github.com/ailienamaggiolo/alphafold_coloring). Transcripts were annotated to the reference dataset by BLAST [59] similarity using blastp, using maximum value for identity and minimum value for E-value, and with the option “-subject_besthit -max_target_seqs 1 -evalue 1e-5” enabled to keep just the best match for the best alignment of each query sequence.

### Expression abundance calculation and visualization

All of the clean reads obtained from mRNA-seq were mapped to the PacBio reference transcript using Bowtie2 [60] (-q --phred33 --sensitive --dpad 0 --gbar 99,999,999 --mp 1,1 --np 1 --score-min L,0,-0.1 -I 1 -X 1000 --no-mixed --no-discordant -p 40 -k 200). We calculated the read count and expression (FPKM, TPM) of transcript with RSEM [61] for each individual. A heatmap of gene expression was drawn using R package pheatmap based on Z-scores-normalized FPKM values. Differential expression gene (DEG) analysis was performed using R package edgeR version 3.36.0 [62]. Lowly expressed genes were discarded using the *filterByExpr* function with default arguments. Raw P-values were BH adjusted for multiple testing. The *prcomp* function of stats packages in R performed the PCA procedure. Pathway enrichment analysis were performed in the clusterProfiler package [63]. Visualizations of DEG intersecting sets through R package ggvenn and UpSetR [64]. Overview bubble plots of the GO (Gene Ontology) and KEGG (Kyoto Encyclopedia of Genes and Genomes) enriched terms were made by R package GOplot [65].

### Electronic supplementary material

Below is the link to the electronic supplementary material.

Supplementary Material 1

Supplementary Material 2

Supplementary Material 3

Supplementary Material 4

Supplementary Material 5

Supplementary Material 6

Supplementary Material 7

Supplementary Material 8

## Data Availability

The raw sequence data reported in this paper have been deposited in the Genome Sequence Archive (GSA) [66] in National Genomics Data Center (NGDC) [67], China National Center for Bioinformation/Beijing Institute of Genomics (CNCB/BIG), Chinese Academy of Sciences (CAS) (PRJCA017042, CRA011075, CRA011260, OMIX004092) that are publicly accessible at https://ngdc.cncb.ac.cn/gsa and https://ngdc.cncb.ac.cn/omix.
