# Supplementary material for: Comparative transcriptomic analysis delineates adaptation strategies of Rana kukunoris toward cold stress on the Qinghai-Tibet Plateau
Source: BMC Genomics. 2024 Apr 12;25:363. doi: 10.1186/s12864-024-10248-8 (PMC11015565; doi:10.1186/s12864-024-10248-8)
Supplement: Supplementary file 8 — Supplementary Material 8 [file 12864_2024_10248_MOESM8_ESM.docx]

**
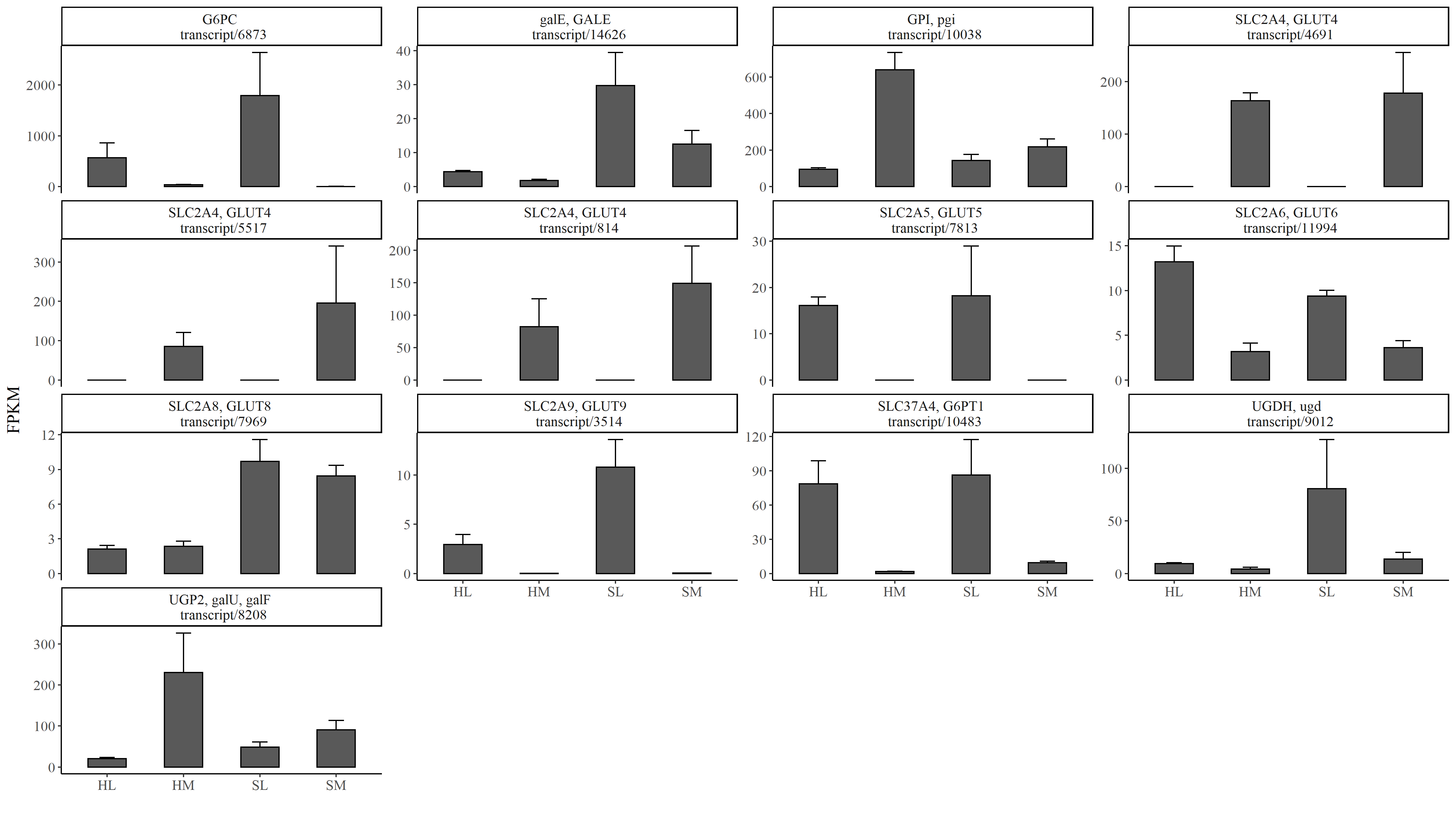
**

Supplementary Figure 1. Expression histogram of glucose-related expression genes in tissue and season of *Rana kukunoris*. The protein name and the transcript ID in sequence file are listed in two lines of text on the top of the subgraph. liver (L), muscle (M), hibernation (H) and spring (S). GPI, pgi: glucose-6-phosphate isomerase [EC:5.3.1.9]; SLC2A6, GLUT6: MFS transporter, SP family, solute carrier family 2 (facilitated glucose transporter), member 6; galE, GALE: UDP-glucose 4-epimerase [EC:5.1.3.2]; SLC2A9, GLUT9: MFS transporter, SP family, solute carrier family 2 (facilitated glucose transporter), member 9; SLC2A4, GLUT4: MFS transporter, SP family, solute carrier family 2 (facilitated glucose transporter), member 4; G6PC: glucose-6-phosphatase [EC:3.1.3.9]; SLC2A5, GLUT5: MFS transporter, SP family, solute carrier family 2 (facilitated glucose/fructose transporter), member 5; SLC2A8, GLUT8: MFS transporter, SP family, solute carrier family 2 (facilitated glucose transporter), member 8; UGP2, galU, galF: UTP--glucose-1-phosphate uridylyltransferase [EC:2.7.7.9]; UGDH, ugd: UDPglucose 6-dehydrogenase [EC:1.1.1.22].


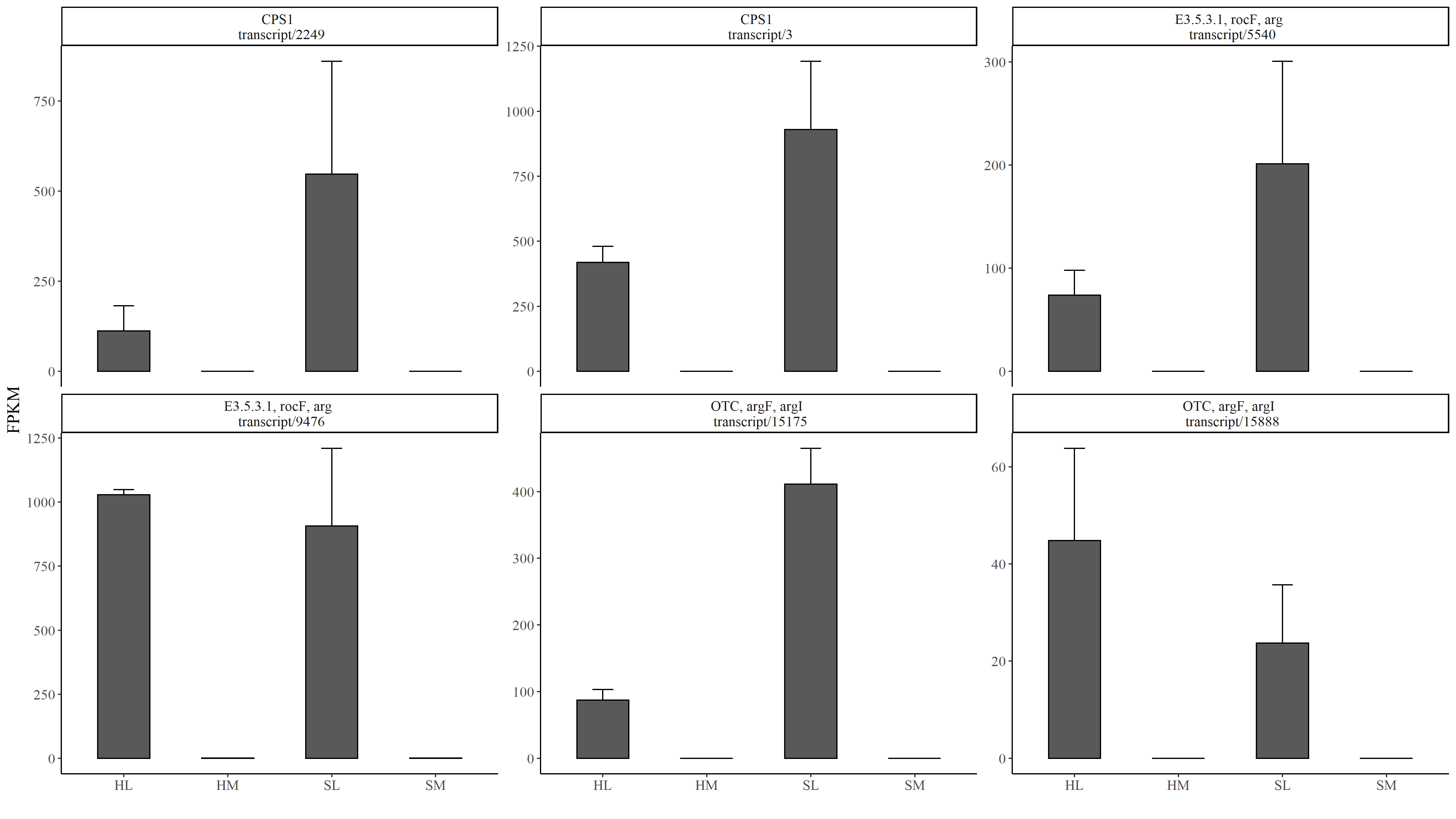


Supplementary Figure 2. Expression histogram of urea-related expression genes in tissue and season of *Rana kukunoris*. The protein name and the transcript ID in sequence file are listed in two lines of text on the top of the subgraph. liver (L), muscle (M), hibernation (H) and spring (S). OTC, argF, argI: ornithine carbamoyltransferase [EC:2.1.3.3]; CPS1: carbamoyl-phosphate synthase (ammonia) [EC:6.3.4.16]; rocF, arg: arginase [EC:3.5.3.1].


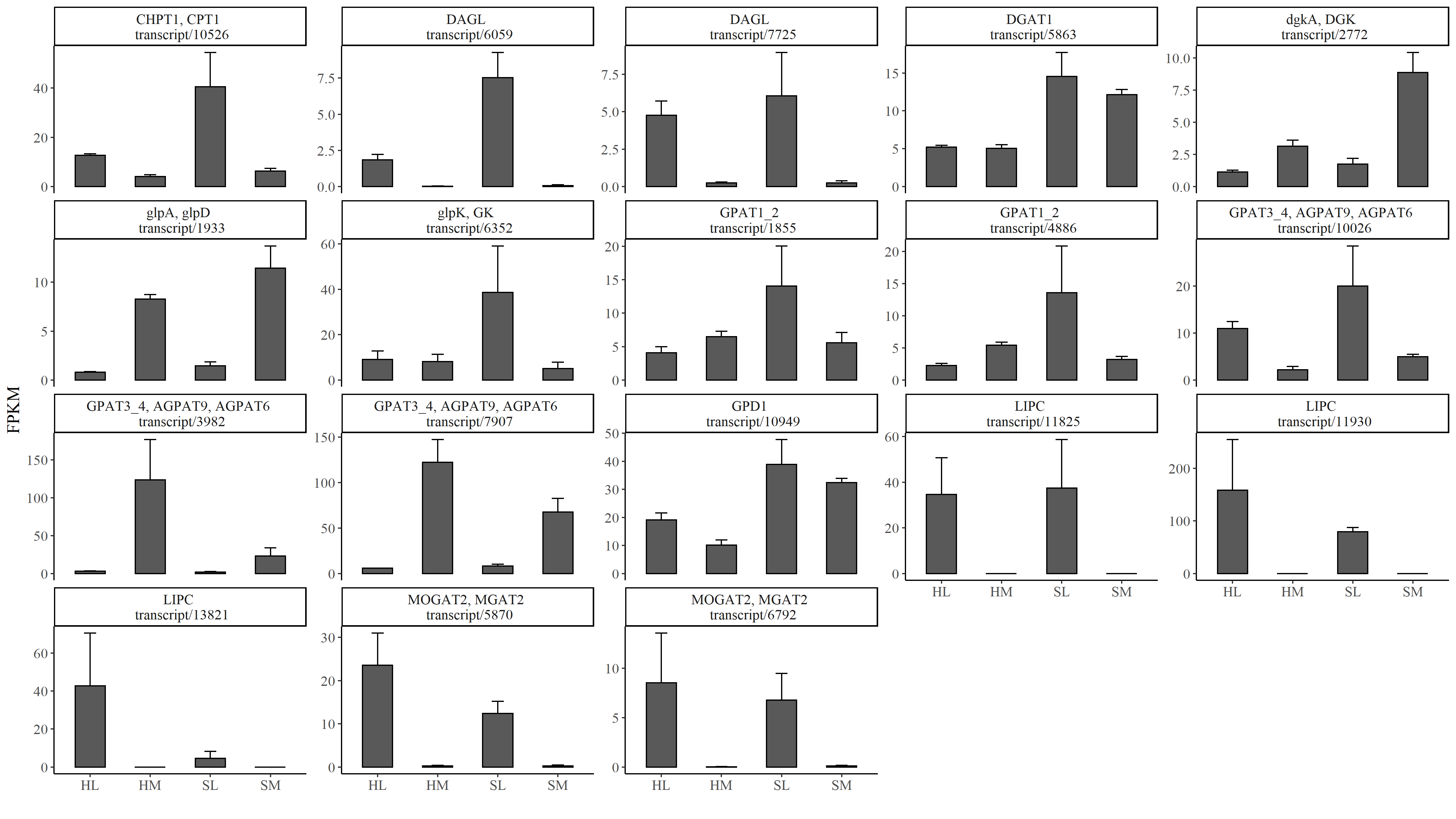


Supplementary Figure 3. Expression histogram of glycerol-related expression genes in tissue and season of *Rana kukunoris*. GPAT3_4, AGPAT9, AGPAT6: glycerol-3-phosphate O-acyltransferase 3/4 [EC:2.3.1.15]; CHPT1, CPT1: diacylglycerol cholinephosphotransferase [EC:2.7.8.2]; GPD1: glycerol-3-phosphate dehydrogenase (NAD+) [EC:1.1.1.8]; LIPC: hepatic triacylglycerol lipase [EC:3.1.1.3]; GPAT1_2: glycerol-3-phosphate O-acyltransferase 1/2 [EC:2.3.1.15]; glpA, glpD: glycerol-3-phosphate dehydrogenase [EC:1.1.5.3]; dgkA, DGK: diacylglycerol kinase (ATP) [EC:2.7.1.107]; DGAT1: diacylglycerol O-acyltransferase 1 [EC:2.3.1.20 2.3.1.75 2.3.1.76]; MOGAT2, MGAT2: 2-acylglycerol O-acyltransferase 2 [EC:2.3.1.22]; DAGL: sn1-specific diacylglycerol lipase [EC:3.1.1.116]; glpK, GK: glycerol kinase [EC:2.7.1.30].


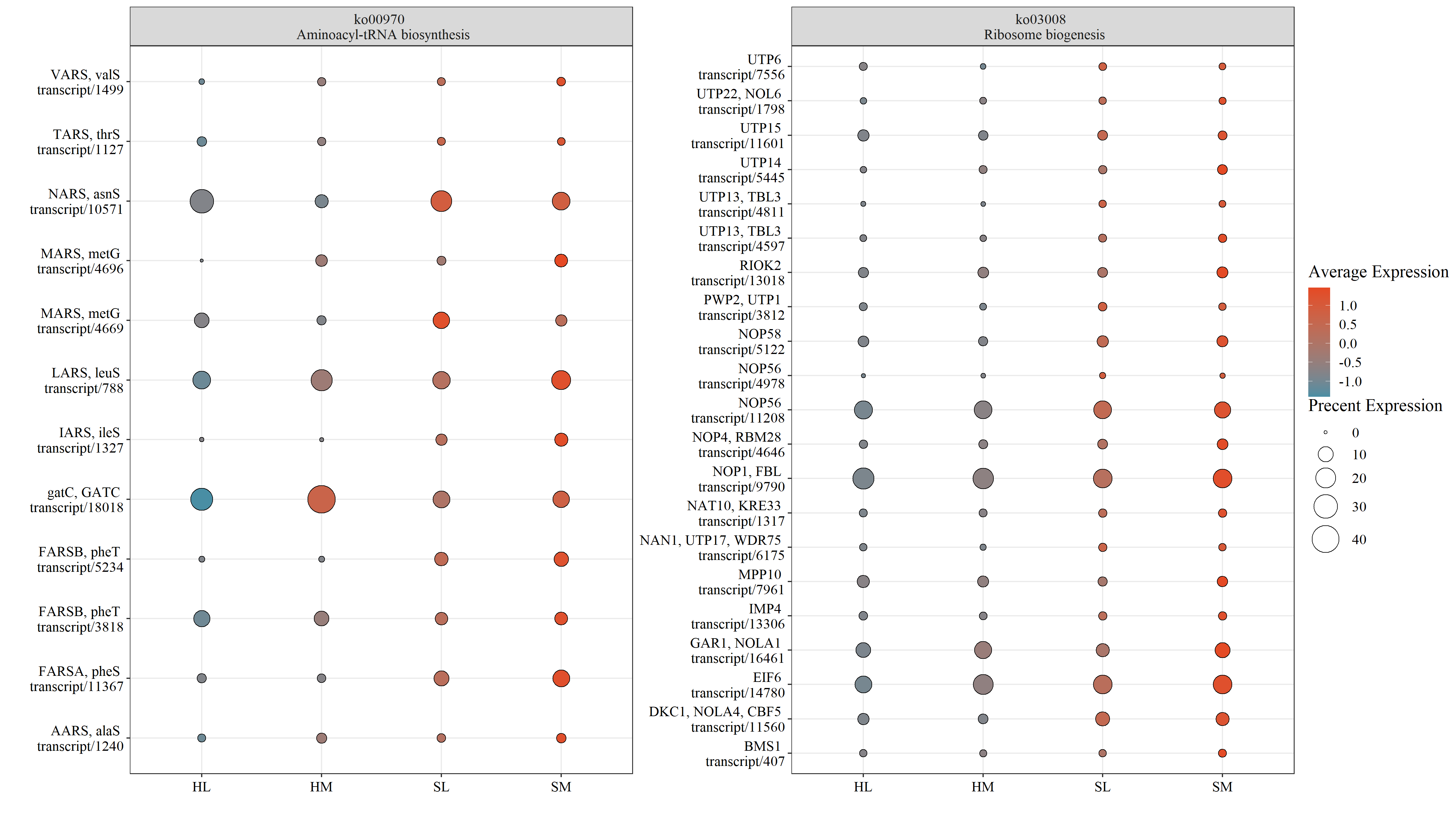


Supplementary Figure 4. Dot plot depicting average and percent expression of genes in down-regulated KEGG pathways in the HL vs SL and HM vs SM. Average expression was z-score FPKM in the four groups. Percent expression was proportion of each transcript FPKM in the term. liver (L), muscle (M), hibernation (H) and spring (S).


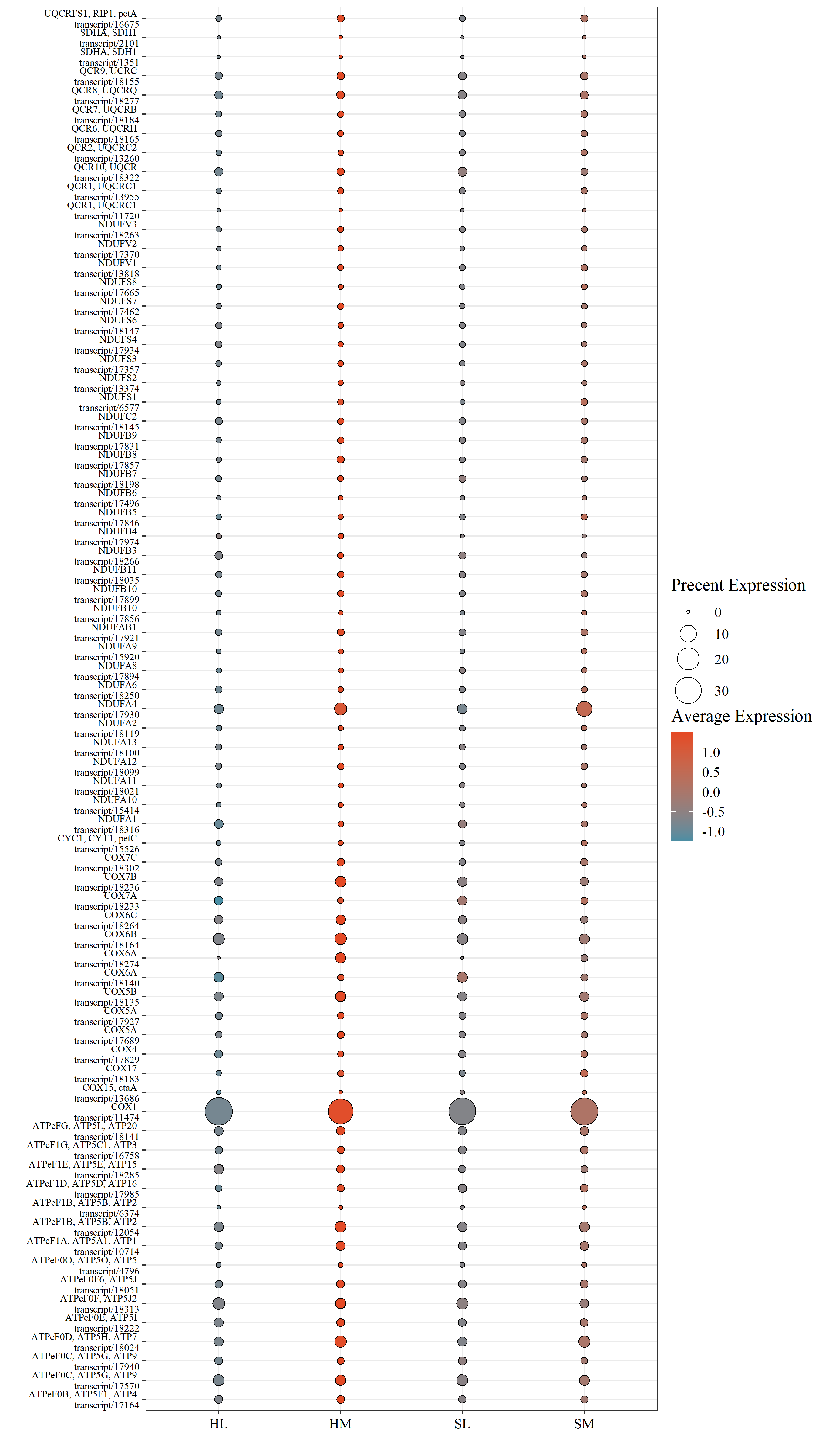


Supplementary Figure 5. Dot plot depicting average and percent expression of genes in the oxidative phosphorylation pathways (ko00190) in the HM vs SM. Average expression was z-score FPKM in the four groups. Percent expression was proportion of each transcript FPKM in the term. liver (L), muscle (M), hibernation (H) and spring (S).


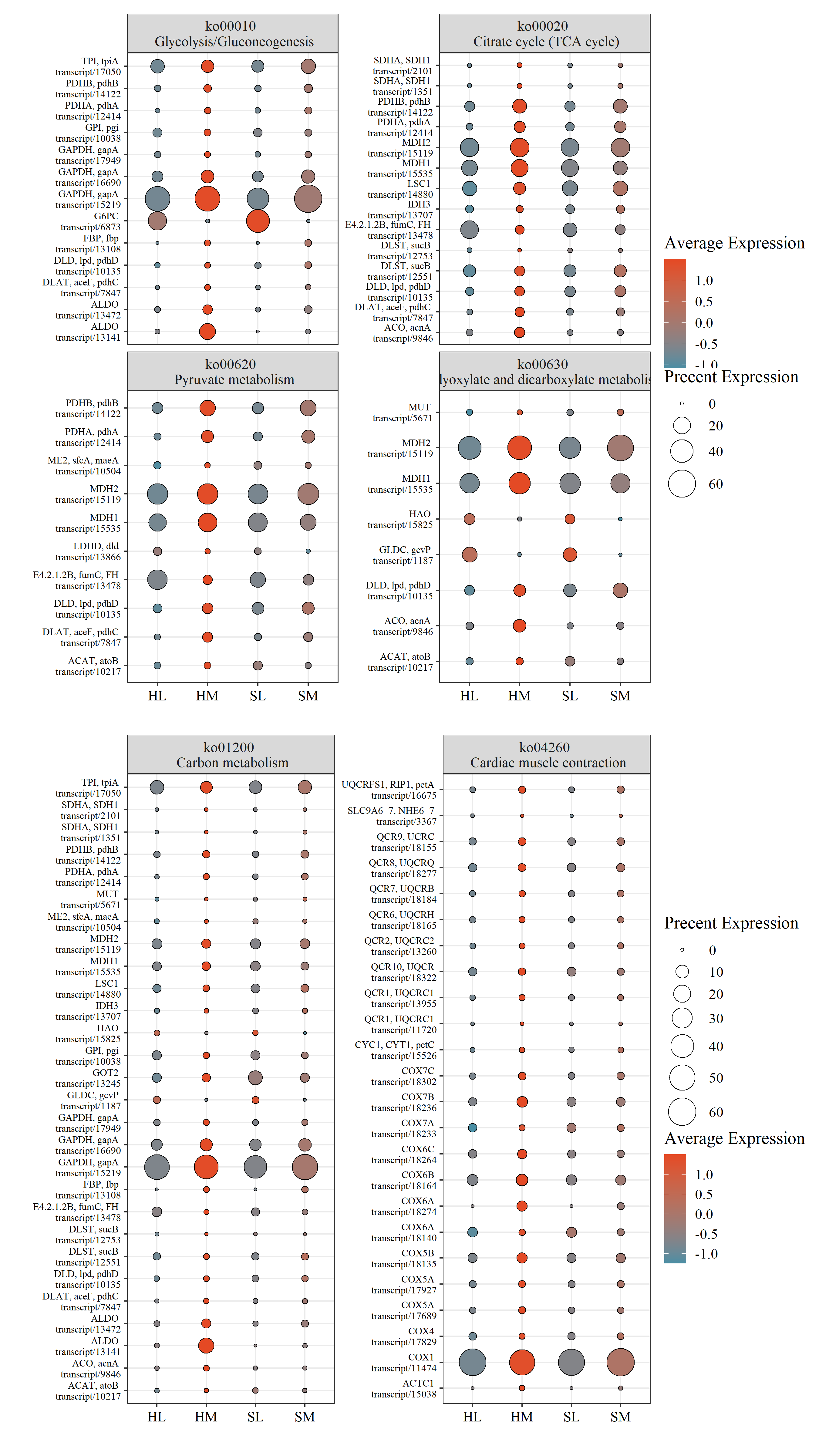


Supplementary Figure 6. Dot plot depicting average and percent expression of genes in up-regulated KEGG pathways in the HM vs SM. Average expression was z-score FPKM in the four groups. Percent expression was proportion of each transcript FPKM in the term. liver (L), muscle (M), hibernation (H) and spring (S).


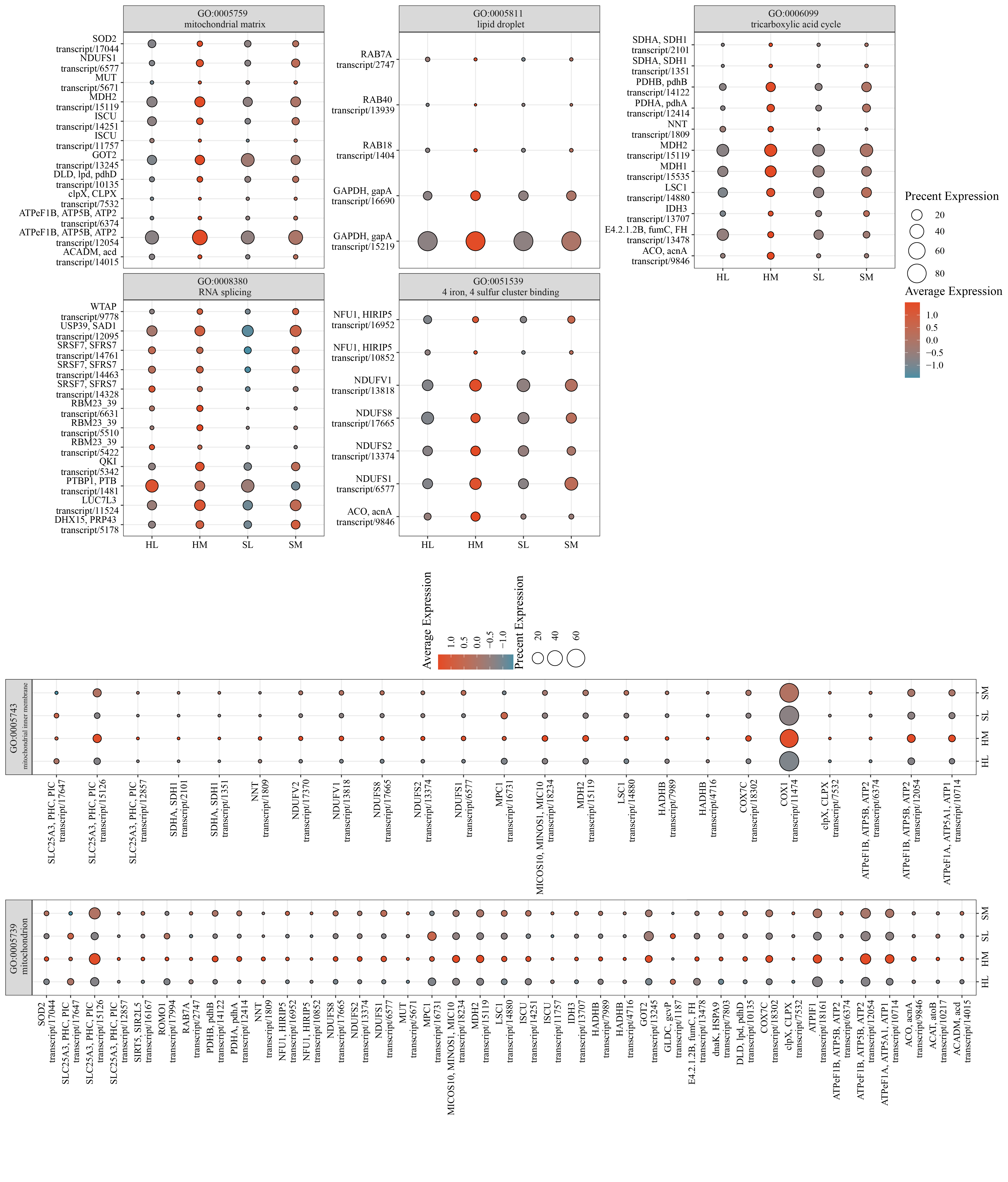


Supplementary Figure 7. Dot plot depicting average and percent expression of genes in the significantly enriched GO terms in the HL vs SL and HM vs SM. Average expression was z-score FPKM in the four groups. Percent expression was proportion of each transcript FPKM in the term. liver (L), muscle (M), hibernation (H) and spring (S).


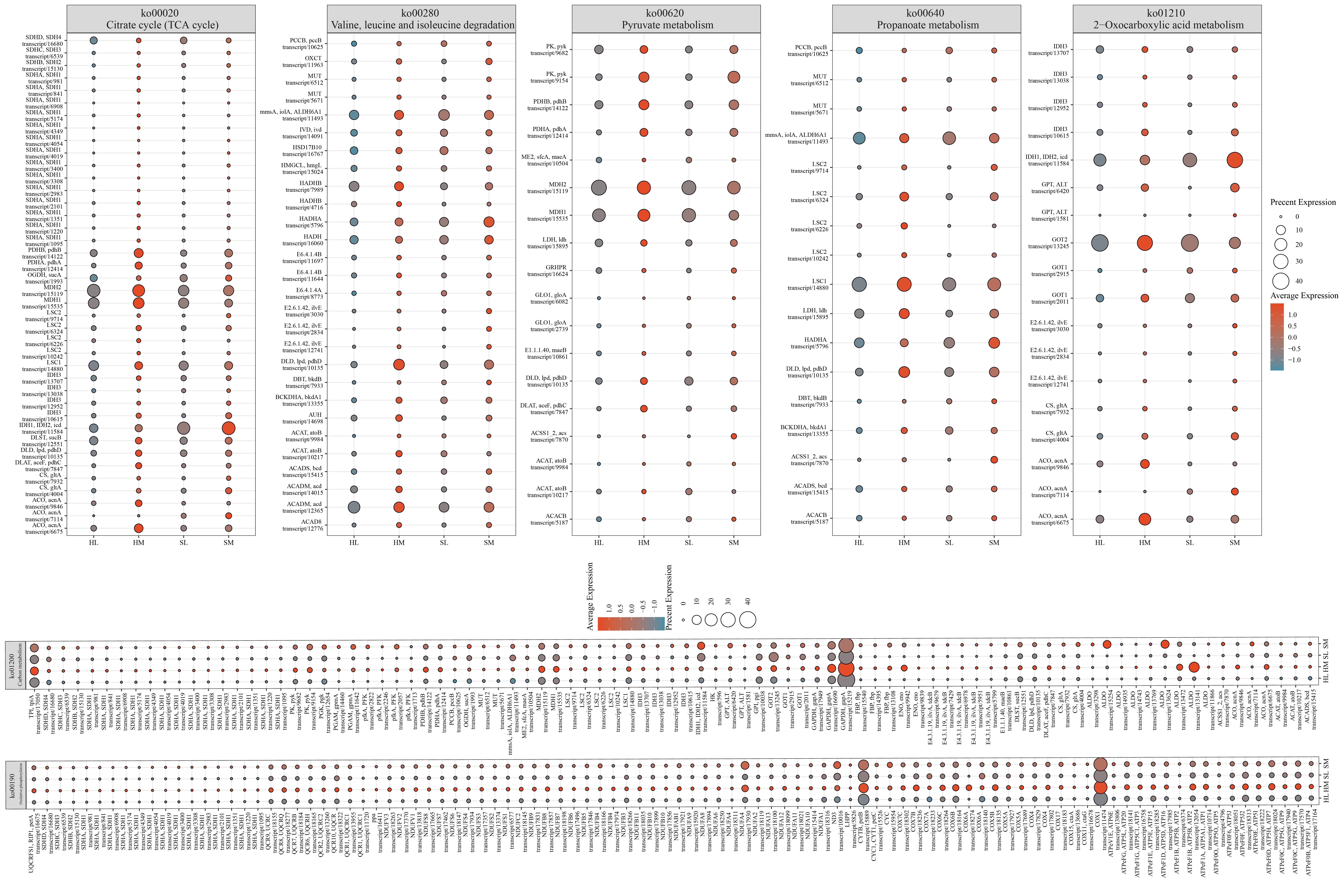


Supplementary Figure 8. Dot plot depicting average and percent expression of genes in the particular down-regulated KEGG pathways in the HL vs HM. Average expression was z-score FPKM in the four groups. Percent expression was proportion of each transcript FPKM in the term. liver (L), muscle (M), hibernation (H) and spring (S).


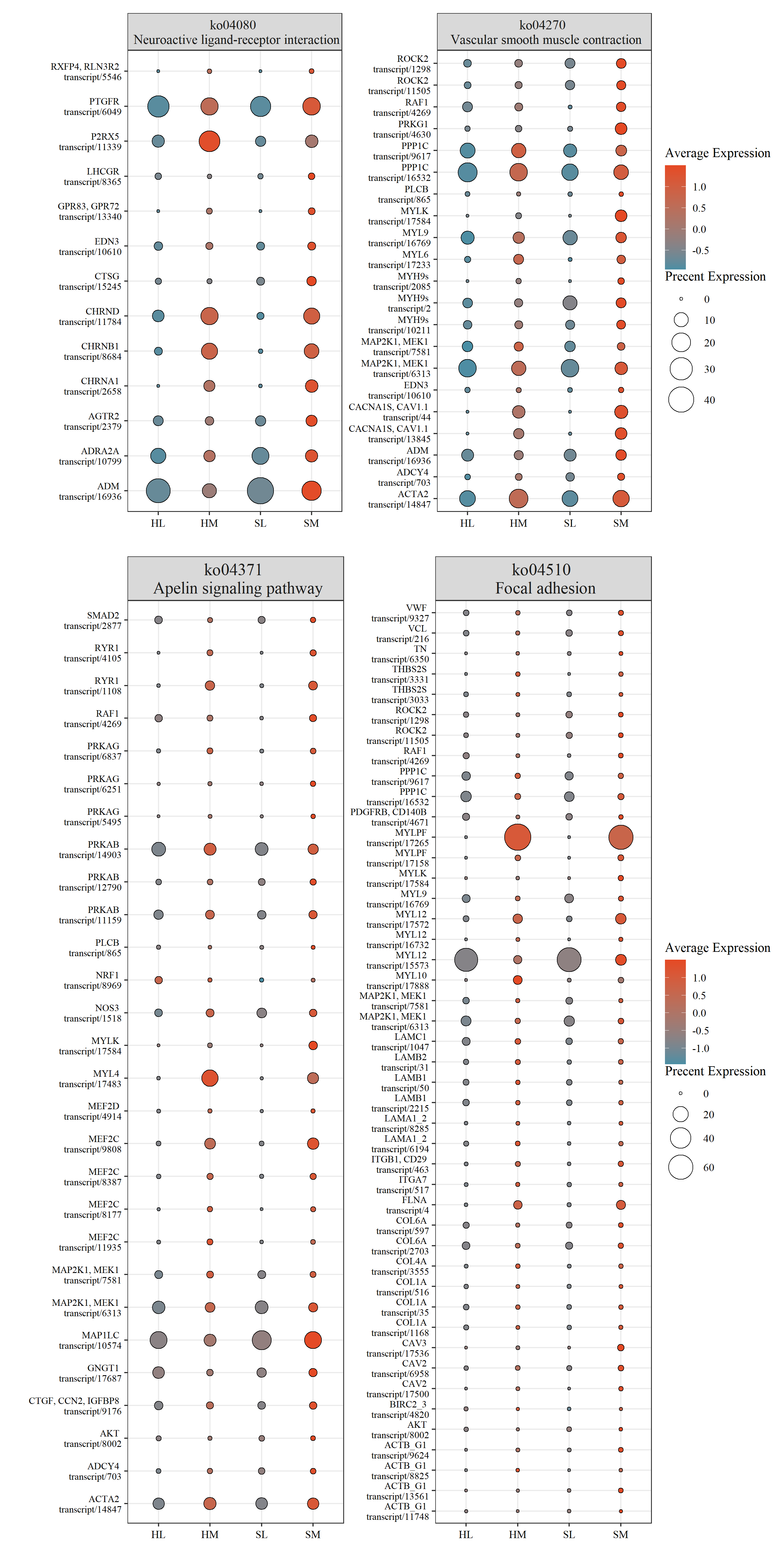


Supplementary Figure 9. Dot plot depicting average and percent expression of genes in the particular down-regulated KEGG pathways in the SL vs SM. Average expression was z-score FPKM in the four groups. Percent expression was proportion of each transcript FPKM in the term. liver (L), muscle (M), hibernation (H) and spring (S).


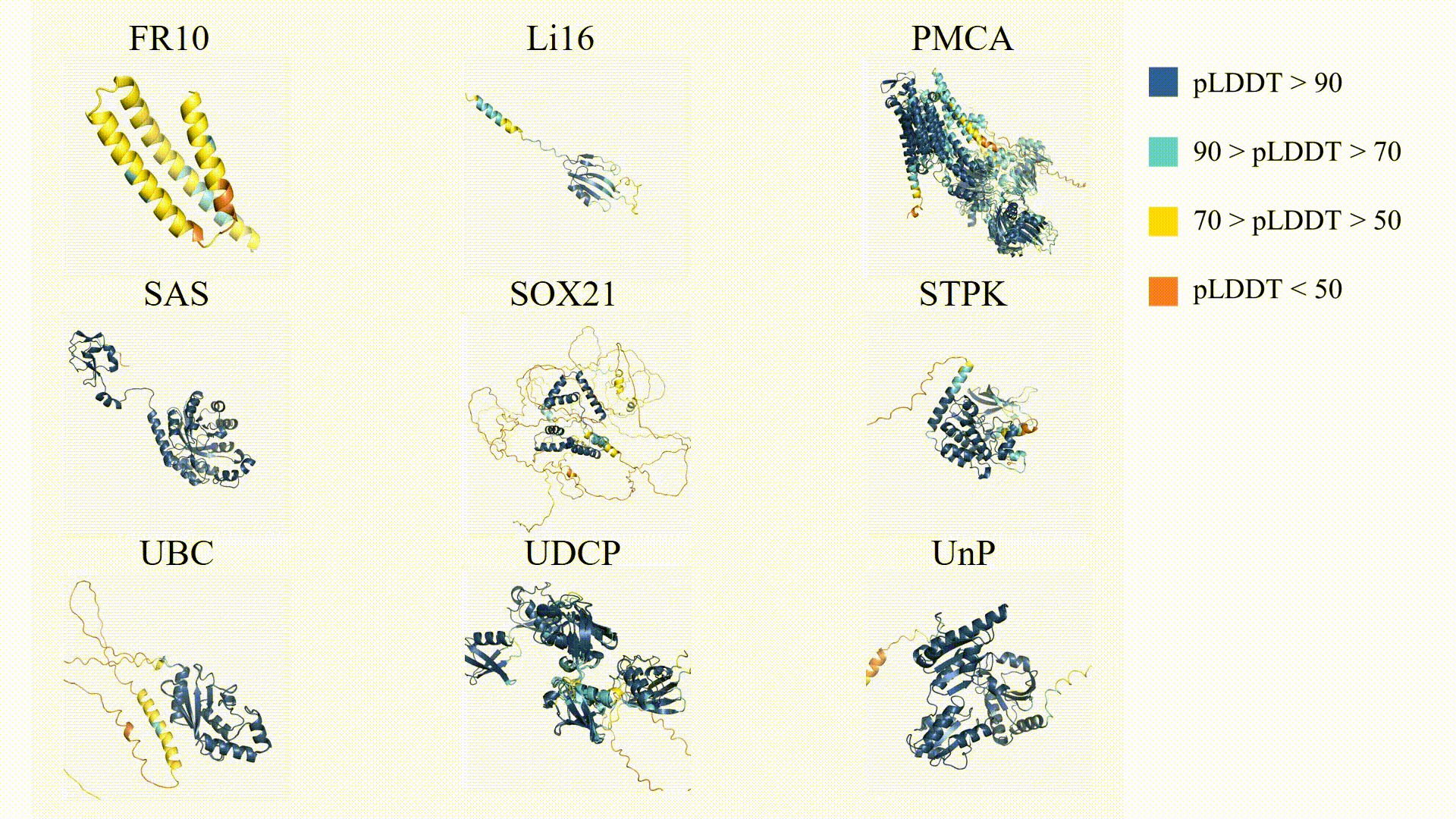


Supplementary Data 1. Predicated three-dimensional structural models of *Rana Kukunoris* putative AFP which show as GIF (graphics interchange format). Models were created using AlphaFold (version 2.0.0) and rendered in PyMOL (version 2.5.0). FR10: a small protein of 90 amino acids with a molecular weight of about 10 kDa; Li16: Freeze-responsive liver protein Li16; PMCA: Plasma membrane calcium ATPase 1b, related; SAS: Sialic acid synthase; SOX21: SOX21 factor; STPK: Serine/threonine-protein kinase mig-15-like; UBC: UBC core domain-containing protein; UDCP: Ubiquitin-like domain-containing protein; Unp: Uncharacterized protein. The pLDDT (predicted local distance difference test) score for each residue serves as the basis for the color scale.
